# Supplementary material for: New Perspectives on the Old Uses of Traditional Medicinal and Edible Herbs: Extract and Spent Material of Persicaria hydropiper (L.) Delarbre
Source: Nutrients. 2024 Oct 3;16(19):3368. doi: 10.3390/nu16193368 (PMC11478433; doi:10.3390/nu16193368)
Supplement: Supplementary file 1 [file nutrients-16-03368-s001.zip › nutrients-3227027-supplementary.pdf]

Supplementary 1

Nutrition, Supplementation and Mental Health Questionnaire

1. The gender with which you identify is

- Female
- Male
- Skip the question

2. How old are you?

- 18-25
- >25-30
- >30-35
- >35-40
- >40-45
- >45-50
- >50-55
- >55-65
- >65
- Skip the question

3. Please choose your answer to the statement: "I have lived in Serbia since I was born."

- Yes
- No
- Skip the question

4. Please choose your answer to the statement: "I currently live in Serbia.“

- Yes
- No
- Skip the question

5. Please choose your answer to the statement: "I consider myself to belong to the following group”:

- Flexeterians - I consume meat in moderation, if I have a choice between meat products and plant products, I will more often choose plant products, fruits, vegetables, mushrooms and/or algae
- Vegans - my diet consists exclusively of food based on plant products, fruits, vegetables and/or algae, mushrooms.
- Omnivores - I rely on a mixed diet, I consume meat, fish as well as plant products, fruits, vegetables, mushrooms, and/or algae and the rest of edible food (dairy products, eggs).
- Ovo-vegetarian - my diet consists of plant products, fruits, vegetables, mushrooms and/or algae, eggs, I do not eat meat, fish, other animal products, dairy products
- Pesco-vegetarians - my diet consists of plant products, fruits, vegetables, mushrooms and/or algae and fish, I do not eat meat, eggs or dairy products
- Lacto-ovo-vegetarians - My diet consists of dairy products and eggs, plant products, fruits, vegetables, mushrooms and/or algae, but I do not eat meat, fish or other meat products

- Lacto-vegetarians - My diet consists of dairy products, plant products, fruits, vegetables, mushrooms and/or algae, but I do not eat meat, egg, fish or other animal-based products
- Skip the question

6. Do you use natural herbal products in the form of teas and/or herbal extracts?

- Yes, often, more than 3 times a week
- Yes, sometimes, 1 or 2 times a week
- Yes, rarely, less than once a week
- Yes, but extremely rarely, in specific situations (when I'm sick or on vacation, or during a certain social event)
- No
- Skip the question

7. Please choose your answer to the statement: "I used the medicinal and edible herb water pepper [lat. *Persicaria hydropiper* (syn. *Polygonum hydropiper*), (papreni lisac in Serbian)]."

- Yes, as a home remedy
- Yes, as a spice and ingredient in meal preparation
- Yes, both as a medicine and as a spice and ingredient in meal preparation
- No
- Skip the question

8. Please choose your answer to the statement: "I used the buckwheat products (lat. *Fagopirum esculentum*)"

- Yes, as a home remedy
- Yes, as an ingredient in meal preparation
- Yes, both as a home remedy and as an ingredient in meal preparation
- No
- Skip the question

9. Please choose your answer to the statement: "I know that water pepper and buckwheat are plants that grow in Serbia."

- Yes
- No
- Skip the question

10. Please choose your answer to the statement: "I know that water pepper and buckwheat are herbs that could contribute to the treatment of depression."

- Yes
- No
- Skip the question

11. Please choose your answer to the statement: "I would rather try a product that contains domestic plants (edible and medicinal plants that grow in Serbia) than other herbal products."

- Yes

- No
- Skip the question

12. Do you know what zero waste culture is?

- Yes
- No
- Skip the question

13. Would you consider introducing into your regular diet functional ingredients or prepared products that are based on bio-agro waste (i.e. spent plant material from tea or extract preparation)?

- Yes
- No
- Skip this question

14. How do you describe your relationship with food?

- I am constantly trying new and different foods
- I am skeptical of new types of food
- Skip this question

15. How do you describe your relationship with food?

- If I don't know what's in the food, whether it's from a reputable restaurant or not, I won't eat it
- If I don't know what's in the food, and the food is from a reputable restaurant, I'll try it
- Skip this question

16. How do you describe your relationship with food?

- I like food from different countries. At dinner, I like to try new food
- I'm afraid to eat things I've never eaten before. I am very picky about the food I eat
- Skip the question

17. Please choose your answer to the statement: "I believe that products containing medicinal herbs and health-promoting bacteria can contribute to the preservation and improvement of the user's mental health."

- Yes
- No
- Skip the question

18. Please choose your answer to the statement: "I would try products that contain medicinal herbs, probiotics and bacteria that promote health and contribute to the preservation and improvement of the user's mental health."

- Yes
- No
- Skip the question

19. Please choose your answer to the statement:"I would try products that contain herbs, psychobiotics and bacteria that promote health, achieve psychobiotic effects in the user and that can be used in the prevention and treatment of depression."

- Yes
- No
- Skip the question

20. Please choose your answer to the statement:"I would try products that contain medicinal herbs, neurobiotics and bacteria that promote health and achieve positive neuroprotective effects in users and that have a positive effect in the prevention and treatment of depression."

- Yes
- No
- Skip the question

21. Please choose your answer to the statement:"I need additional education on the use of plants in the diet as a preventive measure of depression, on psychobiotics (syn. neurobiotics) and probiotics."

- Yes
- No
- Skip the question

ОПШТА БОЛНИЦА ЋУПРИЈА

Бр. 10332/1/4
12. 09. 2023.

Миодрага Новаковића 78, 35230 Ћуприја, Србија
Централа (+381 35) 8470 775 / Тел/Факс (+381 35) 8474 306
Директор (+381 35) 8471 503
МБР 17729969 / ПИБ 105541752 / Шифра делатности 8610
Рачун 840-787661-65 / Сопствени приходи 840-773667-46
www.bolnicacuprija.com / bolnicacuprija@mts.rs

На основу одредаба Закона о здравственој заштити Републике Србије („Сл.гласник РС“, БР.25/2019), поступајући у складу са Начелима добре клиничке праксе , Етички одбор Опште болнице Ћуприја у саставу:

1.Др Слободанка Ђорђевић, специјалиста психијатрије, председник,

2.Др Слађана Старчевић, члан

3.Периша Поповић, дипл.правник у РФЗО-Испостава Ћуприја, члан и

4.Душан Катић, дипл.правник, члан

на седници одржаној дана 12.09.2023.год. размотрио је молбу др сци.мед.Петра Војводића, бр.9606/1 од 22.08.2023.год.

О Д Л У К У

Одобрава се спровођење истраживања за потребе израде научног рада на начин и под условима наведеним у молби и документацији др сци мед. Петра Војводића заведен под бројем 9606/1 од 22.08.2023.год.

Наслов научног рада: „Испитивање сензорне прихватљивости и општег интересовања за млечне и немлечне производе ферментисане пробиотским природним изолатима бактерија млечне киселине на узорку модерних потрошача укључујући и пацијенте оболеле од депресивних поремећаја“ у Служби психијатрије Опште болнице Ћуприја.

Место спровођења истраживања: Служба психијатрије Општа болница Ћуприја.

Истраживачи: др сци.мед.Петар Војводић, др био. Марина Јовановић- научни сарадник, др био. Дина Тењи-научни сарадник, др Јелена Миочиновић-редовни професор

Председник Етичког одбора
Опште болнице Ћуприја
Др Слободанка Ђорђевић

Official translation

Beginning of translation

STAMP

No. 10332/1/4

9/12/2023

Ćuprija General Hospital

Based on the provisions of the Law on Health Protection of the Republic of Serbia (Official Gazette of the Republic of Serbia No. 25/2019) acting in accordance with the Principles of Good Clinical Practice, the Ethics Committee of the General Hospital of Ćuprija, consisting of:

1. Dr Slobodanka Đorđević, specialist in psychiatry, chairman
2. Dr Slađana Starčević, member
3. Periša Popović, attorney in law, RFZO Branch Ćuprija, member and
4. Dušan Katić, attorney in law, member

At the session held on September 12, 2023, the request of dr.sci.med. Petar Vojvodić, no. 9606/1 dated 8/22/2023 was considered.

Decision

The conduct of research for the purposes of creating a scientific study is approved in the manner and under the conditions specified in the application and documentation of Dr. Sci Med. Petar Vojvodić filed under number 9606/1 dated 8/22/2023

Scientific research under the title: "Examination of sensory acceptability and general interest in dairy and non-dairy products fermented with probiotic natural isolates of lactic acid bacteria on a sample of modern consumers, including patients suffering from depressive disorders" in the Service of the Psychiatric Hospital in Ćuprija.

Place of conducting research: Department of Psychiatry, General Hospital Ćuprija.

Researchers: dr.sci.med. Petar Vojvodić, dr bio. Marina Jovanović- research associate, dr. bio. Dina Tenji- research associate, dr Jelena Miočinović- full professor.

Chairman of the ethics committee of the General Hospital of Ćuprija

Signature

Dr Slobodanka Đorđević

End of translation

Date 9/2/2024

ILS PROVIDA FUTURI d.o.o. Zrenjanin

Boris Kirćanski
Principal
ILS Provida Futuri
